# Synergid cell calcium oscillations refine understanding of FERONIA/LORELEI signaling during interspecific hybridization

**DOI:** 10.1007/s00497-023-00483-6

**Published:** 2023-11-07

**Authors:** Nathaniel Ponvert, Mark A. Johnson

**Affiliations:** 1 Department of Plant Sciences, University of Arizona, Tucson, AZ 85721 USA; 2 Department of Molecular Biology, Cell Biology, and Biochemistry, Brown University, Providence, RI 02912 USA

**Keywords:** Signaling, Hybridization, Calcium, Speciation, Pollen, Synergid

## Abstract

**Key message:**

Pollen tubes from closely related species and mutants lacking pollen tube MYB transcription factors are able to initiate FER/LRE-dependent synergid cell calcium oscillations.

**Abstract:**

Reproductive isolation leads to the evolution of new species; however, the molecular mechanisms that maintain reproductive barriers between sympatric species are not well defined. In flowering plants, sperm cells are immotile and are delivered to female gametes by the pollen grain. After landing on the stigmatic surface, the pollen grain germinates a polarized extension, the pollen tube, into floral tissue. After growing via polar extension to the female gametes and shuttling its cargo of sperm cells through its cytoplasm, the pollen tube signals its arrival and identity to synergid cells that flank the egg. If signaling is successful, the pollen tube and receptive synergid cell burst, and sperm cells are released for fusion with female gametes. To better understand cell–cell recognition during reproduction and how reproductive barriers are maintained between closely related species, pollen tube-initiated synergid cell calcium ion dynamics were examined during interspecific crosses. It was observed that interspecific pollen tubes successfully trigger synergid cell calcium oscillations—a hallmark of reproductive success—but signaling fails downstream of key signaling genes and sperm are not released. This work further defines pollen tube–synergid cell signaling as a critical block to interspecific hybridization and suggests that the FERONIA/LORELEI signaling mechanism plays multiple parallel roles during pollen tube reception.

**Supplementary Information:**

The online version contains supplementary material available at 10.1007/s00497-023-00483-6.

## Introduction

Successful reproduction and propagation of a species require that a pair of gametes unite and fuse to form a zygote. When gametes from two different species unite, the resulting zygote is often inviable or sterile, posing a postzygotic barrier to interspecific hybridization (Moyle et al. 2014). This outcome is suboptimal for mating partners because resources used to produce gametes do not result in viable, fertile offspring. Consequently, there is evolutionary pressure favoring mechanisms that act either before or after mating, to prevent fusion with gametes of different species (Moyle et al. 2014). These mechanisms are particularly important for plants, which have less control over the partners with whom they mate.

One of the best-studied mechanisms operating as a prezygotic barrier to interspecific mating in plants is the rapid evolution of flower color that reinforces same-species mating by ensuring animal pollinators deliver pollen to the stigma of the same species (Hopkins and Rausher 2012). The present study focuses on barriers that function downstream of pollinator activity to prevent interspecific fertilization in cases where pollen from one species has already been delivered to (in animal pollination systems) or has already landed on (in wind pollinated systems) the stigma of a different species.

**Fig. 1:**
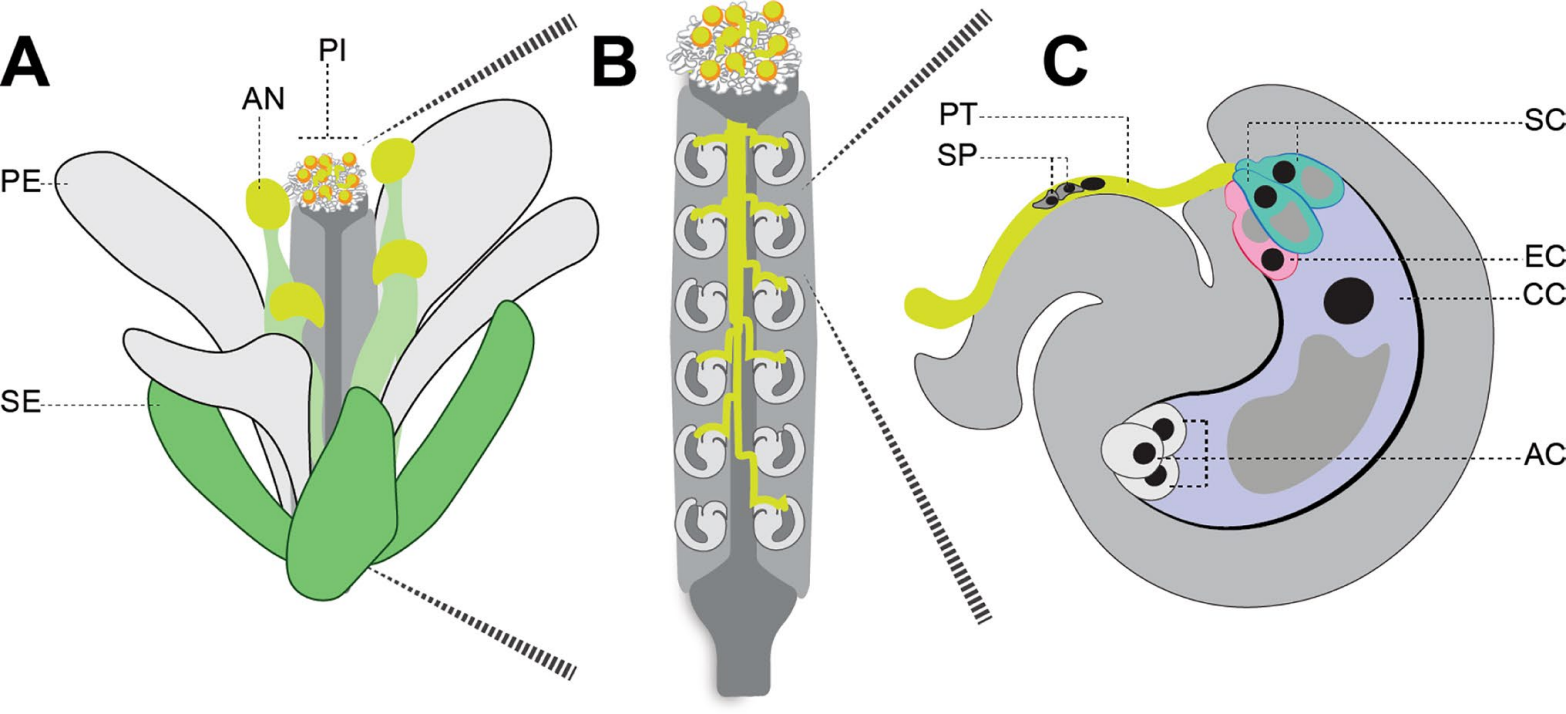
The pollen tube delivers immotile sperm to female gametes. **A** A diagram of an *Arabidopsis thaliana* flower showing sepals (SE), petals (PE), anthers (AN), and pistil (PI). **B** A cutaway diagram of the pistil showing pollen tubes growing toward female gametes within ovules. **C** A diagram of the ovule, showing the pollen tube (PT), containing sperm cells (SP), approaching the synergid cells (SC) that flank the egg (EC) and sit in front of the larger central cell (CC), and distal antipodal cells (AC)

The pollen grain consists of three cells: two sperm cells, which develop within the cytoplasm of the larger pollen vegetative cell. After landing on the stigmatic surface, pollen grains hydrate and germinate a pollen tube that grows via polarized tip extension through the transmitting tract of the pistil tissue, shuttling the cargo of two immotile sperm cells within their cytoplasm, before emerging onto the interior placental surface of the ovary chamber. The female gametes (the egg cell and the central cell) develop in ovules (Fig. 1A, B) which are positioned within the ovary chamber. In addition to female gametes, ovules also contain egg-flanking synergid cells and distal antipodal cells (Fig. 1C). The targeted growth of the pollen tube tip toward the female gametes relies upon chemoattractants released by the synergid cells (Takeuchi and Higashiyama 2012; Zhong et al. 2019).

Failure of any step during pollen–pistil interactions (Dresselhaus et al. 2016; Higashiyama and Yang 2017; Johnson et al. 2019) results in infertility and can therefore be the focus of selection leading to mechanisms that either actively block interspecific pollen (Bedinger et al. 2011), or that passively disfavor interspecific pollen relative to pollen of the same species (Hogenboom 1975). It has been observed during crosses between *Arabidopsis thaliana* and distantly related species that the earliest event in pollen–pistil interactions, adherence of pollen to stigma, can prevent interspecific fertilization, where the adherence of interspecific pollen was found to be weaker than that of same-species pollen (Zinkl et al. 1999). Further, the ability of pollen tubes to maintain viability while extending through pistil tissue is actively blocked during interspecific crosses when the female parent is a self-incompatible member of the Solanaceae (Li and Chetelat 2010). In dicotyledonous plants, pollen tubes respond to synergid cell-secreted guidance cues (LURE proteins) that lead the extending tip toward the female gametes (Okuda et al. 2009; Takeuchi and Higashiyama 2012). In monocots, the same function has been ascribed to a different small secreted protein (EA1, (Marton et al. 2005)). In both cases, it has been found that pollen tube attractants preferentially attract pollen tubes of the same species (Marton et al. 2012; Takeuchi and Higashiyama 2016; Wang et al. 2016; Zhong et al. 2019). Each of these observations is reflective of the evolutionary pressure acting on this system to favor same-species mating partners over interspecific hybrids.

The synergid cells (Fig. 1) are the final gatekeepers separating sperm and female gametes. If the interaction between the pollen tube and receptive synergid cell is successful, both will rupture resulting in the release of two sperm cells at the site of fusion with female gametes ((Huck et al. 2003; Rotman et al. 2008), Fig. 1C). It has been observed during interspecific crosses that pollen tube and synergid cell rupture fails, providing evidence that this last stage of pollen-pistil interactions can also present a prezygotic barrier to interspecific fertilization (Escobar-Restrepo et al. 2007; Leydon et al. 2014; Galindo-Trigo et al. 2020; Williams et al. 1986).

Genetic analysis (Huck et al. 2003; Kessler et al. 2010; Leydon et al. 2013; Rotman et al. 2008; Capron et al. 2008; Tsukamoto et al. 2010; Liang et al. 2013) has defined a signaling pathway that is required for pollen tube–synergid rupture and sperm release in *Arabidopsis*. Loss of function of the synergid-expressed FERONIA (FER) receptor-like kinase (Huck et al. 2003; Rotman et al. 2008) or its co-receptor (Li et al. 2015; Liu et al. 2016) the glycosylphosphatidylinositol (GPI)-anchored protein LORELEI (LRE, (Capron et al. 2008; Tsukamoto et al. 2010)) results in reduced pollen tube–synergid rupture and a corresponding reduction in fertility. Loss of the synergid-expressed multipass-transmembrane protein NORTIA (NTA) also results in reduction of pollen tube–synergid rupture. It has been shown that NTA functions at the interface between pollen tube and synergid cell as a calcium ion channel, and its relocalization from the endomembrane system to the point of pollen tube contact occurs downstream of FERONIA and may be involved in determining pollen tube suitability via competitive binding between pollen tube ligands and activity of its auto-repressive calmodulin domain (Kessler et al. 2010; Gao et al. 2022). Furthermore, it has been found that double mutants lacking HERCULES (HERK) and ANJEA (ANJ), members of the same malectin-domain containing *Catharanthus roseus* RLK-1 Like Kinase (CrRLK1L) family as FERONIA, and proteins that have been found to bind FERONIA in vitro, phenocopy the sperm release and pollen tube coiling defects observed during crosses with *feronia* loss of function mutants, but do not affect FER-dependent establishment of reactive oxygen species (ROS) maxima at the pollen tube–synergid cell interface (Galindo-Trigo et al. 2020). Taken together, these data suggest that FERONIA plays multiple, distinct roles at different stages of pollen tube–synergid cell signaling. Pollen tube-expressed genes are also important for pollen tube–synergid rupture, as loss of function of pollen tube-expressed MYB transcription factors (*myb97, myb101, and myb120*) and consequent reduced expression of their transcriptional targets also reduces pollen tube–synergid rupture and reduces fertility (Leydon et al. 2013, 2017; Liang et al. 2013).

Interestingly, the *fer*, *lre*, *nta,* and *myb* mutant phenotypes, where pollen tubes fail to release sperm and instead continue to grow within the ovule, closely resemble the defects observed during interspecific crosses of *Arabidopsis* (Escobar-Restrepo et al. 2007; Leydon et al. 2014) and *Rhododendron* (Williams et al. 1986). These observations suggest that FER, LRE, NTA, and MYB-dependent pollen tube genes could be at the core of a prezygotic barrier to interspecific hybridization at the last stage of plant reproduction before gametes are released.

Analysis of genetically encoded calcium ion sensors has shown that contact between the pollen tube tip and the synergid cell of *Arabidopsis thaliana* initiates calcium ion oscillations that persist for approximately 45 min before pollen tube–synergid rupture causes a dramatic final increase in calcium concentration concurrent with sperm release (Denninger et al. 2014; Ngo et al. 2014). The pattern of synergid cell calcium oscillations provides a molecular signature for the progression of pollen tube–synergid interactions and reflects the distinct roles that FER/LRE and NTA play during pollen tube–synergid signaling. While loss of any of the three genes results in failure of pollen–synergid rupture, only loss of FER or LRE completely abrogates synergid calcium oscillations; loss of NTA resulted in aberrant calcium ion oscillations (Ngo et al. 2014). Moreover, loss of FER and LRE results in higher rates of failure of pollen tube–synergid rupture than does loss of NTA.

To more clearly define the defects in molecular signaling events during interspecific pollen tube–synergid cell interactions, patterns of synergid cell calcium ion oscillations during interspecific crosses were analyzed and compared with patterns of synergid cell calcium oscillations observed during same-species crosses between lines lacking key signaling genes. It was found that while interspecific pollen tubes fail to rupture, they are still able to elicit synergid cell calcium ion oscillations in their interspecific mating partners. Moreover, as is the case in same-species crosses within *Arabidopsis thaliana*, synergid oscillations induced by interspecific pollen tubes required FER and LRE activity. These results suggest that species-level identity, critical for pollen tube–synergid rupture, is communicated through a mechanism other than initiation of FER/LRE-dependent synergid cell calcium ion oscillations.

It was also observed that, in contrast to the female *fer* and *lre* mutants that lack calcium ion oscillation responses even during same-species crosses, male *myb* triple-mutant pollen tubes were able to elicit calcium ion oscillations in female cells despite an ultimate failure of pollen tube–synergid rupture. These results are similar to what was observed during interspecific pollination, suggesting that MYB-dependent genes may act downstream of FER/LRE and/or independently of FER/LRE-dependent calcium ion signaling, and may define a portion of the pollen tube’s species-level genetic identity.

## Results

### Same-species pollen tubes induce FER/LRE-dependent synergid cell calcium ion oscillations

**Fig. 2:**
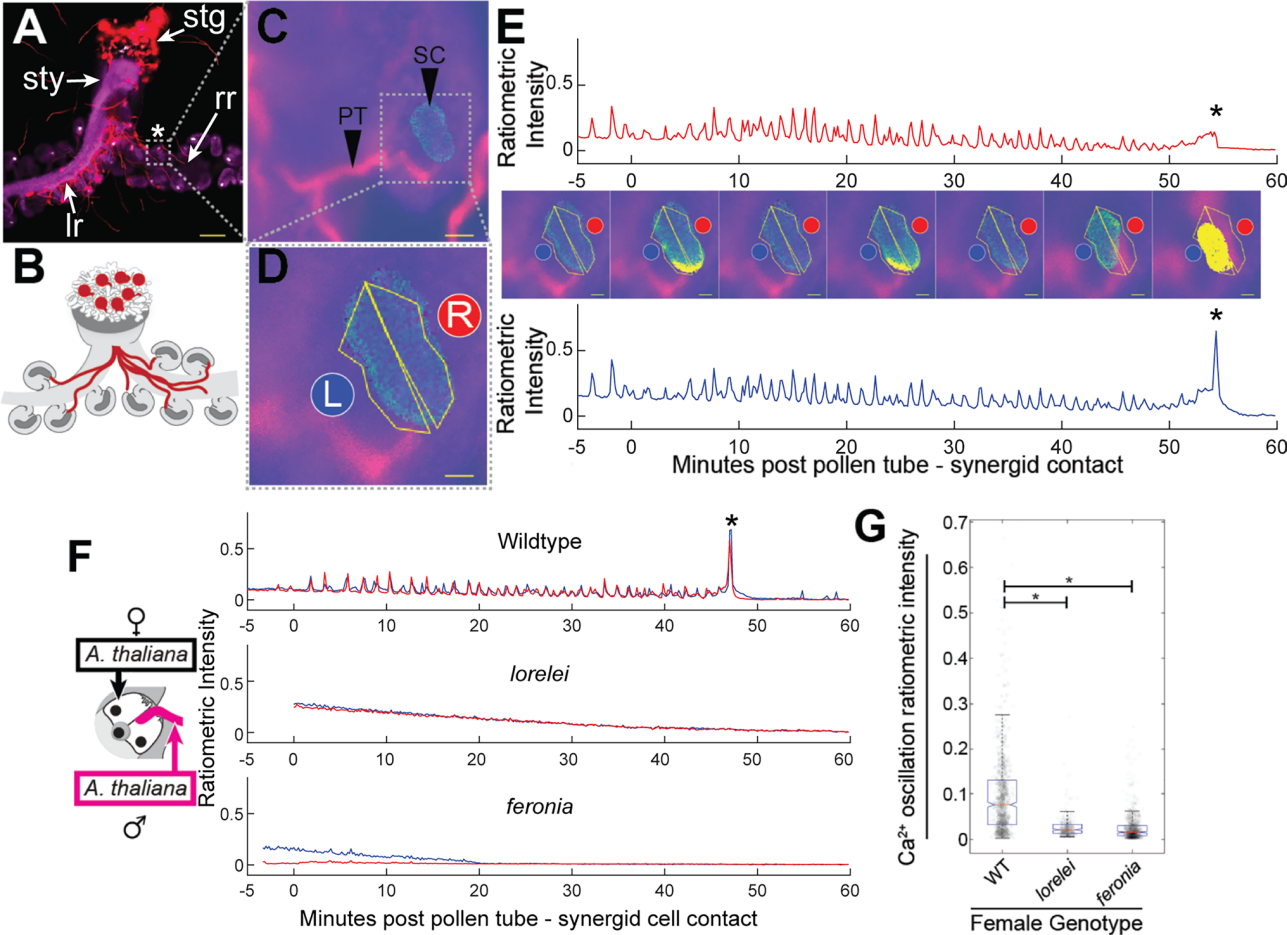
Live imaging of calcium ion oscillations during pollen tube–synergid interactions. **A** A live-dissection approach splits the transmitting tract tissue apart from the bottom up, allowing for single-cell resolution time lapse imaging. Scale bar: 200 μm. stg = stigma, sty = style, lr = left replum, rr = right replum, asterisk = ovule in panel “C”. **B** A cartoon representation of the dissection approach in A. **C** An example image showing a red-fluorescent pollen tube (LAT52:dsRED, PT) approaching synergid cells expressing MYB98:YC3.60 (SC). Scale bar: 20 μm. **D** The left and right synergid cells (L and R) with the regions-of-interest used for measurement of calcium ion oscillations overlaid in yellow. Scale bar: 8 μm. **E** Traces of ratiometric intensity as measured from the right (top graph) and left (bottom graph) synergid cells shown in D with representative frames displayed in the middle. Scale bar: 8 μm. time point 0 indicates the moment of pollen tube–synergid cell contact. Pollen tube–synergid cell burst is indicated with black asterisk. **F** Example traces (additional traces are presented in the supplement) from crosses onto wild-type, *lre*, and *fer* mutant females using wild-type *Arabidopsis thaliana* pollen. **G** Plot of ratiometric intensity of individual calcium ion oscillations (each point) observed during crosses onto female genotypes (wild type, *n* = 10 traces, *n* = 871 peaks; *lre,* *n* = 10 traces, *n* = 239 peaks; *fer,* *n* = 10 traces, *n* = 672 peaks). Median value denoted by notch. 95% confidence interval about the median is denoted by notches. Box defines interquartile range. Length of whiskers is calculated as 1.5*IQR. Statistical significance (*) calculated using analysis of variance (ANOVA, α = 0.05)

To begin the investigation into interspecific interactions, same-species pollen tube–synergid interactions were analyzed, as was done previously (Iwano et al. 2012; Denninger et al. 2014; Borg et al. 2014; Ngo et al. 2014) (Fig. 2, Supplementary Fig. 1A, Supplementary Video 1). A live-dissection approach (Fig. 2A–D, Supplementary Video 13) that allowed imaging interactions between pollen tubes (expressing *LAT52:dsRED*) and synergid cells expressing the FRET-based calcium ion sensor Yellow Cameleon 3.6 (*MYB98:YC3.60*) by confocal microscopy was used. In three out of ten replicates of same-species crosses between wild-type mating partners (Supplementary Fig. 1A, traces number 5, 7, and 8), the orientation of the dissected ovules was such that regions of interest distinguishing the two synergid cells could be defined (as in Fig. 2D, E). In such cases, it was possible to observe calcium oscillations in both synergid cells that commenced at pollen tube arrival and ended when the receptive synergid cell burst (Fig. 2E, Right, R). A concurrent peak of ratiometric intensity, representing calcium influx into the remaining synergid (Fig. 2E, Left, L), was also observed, consistent with previously published data (Denninger et al. 2014; Ngo et al. 2014). In the remaining seven replicates, the orientation of the ovule left the synergid cells overlaid with one another with respect to observation, and for these replicates the calcium ion oscillations of both overlayed synergid cells were collected together. The frequency of oscillations was roughly one peak/minute during the period of pollen tube–synergid interaction (Fig. 2E), which varied in duration from 16 to 58 min.

Also consistent with previous reports (Ngo et al. 2014), it was observed that pollen tube–synergid cell rupture did not occur during interactions between wild-type pollen tubes and *lre* or *fer* mutant ovules and that calcium ion oscillations in *fer* and *lre *mutant synergid cells were severely attenuated (Fig. 2F, Supplementary Fig. 1B, C, Supplementary Videos 2, 3). Furthermore, the intensity of oscillations observed in *lre-* and *fer* mutant synergids was found to be statistically significantly reduced as compared to wild-type (Fig. 2G).

### Interspecific pollen tubes elicit FER/LRE-dependent calcium ion oscillations in synergid cells of different species

Next, it was asked whether calcium ion oscillations occur in synergid cells receiving interspecific pollen tubes. *Arabidopsis thaliana* was used as female and *Arabidopsis lyrata* and *Olimarabidopsis pumila* were used as pollen donors, as they are predicted to be diverged ~ 4–10 million years ago (MYA) and ~ 10–14 MYA from *A. thaliana* respectively, thereby serving as representatives of closely and more distantly related species (Hall et al. 2002; Yogeeswaran et al. 2005; Beilstein et al. 2010).

**Fig. 3:**
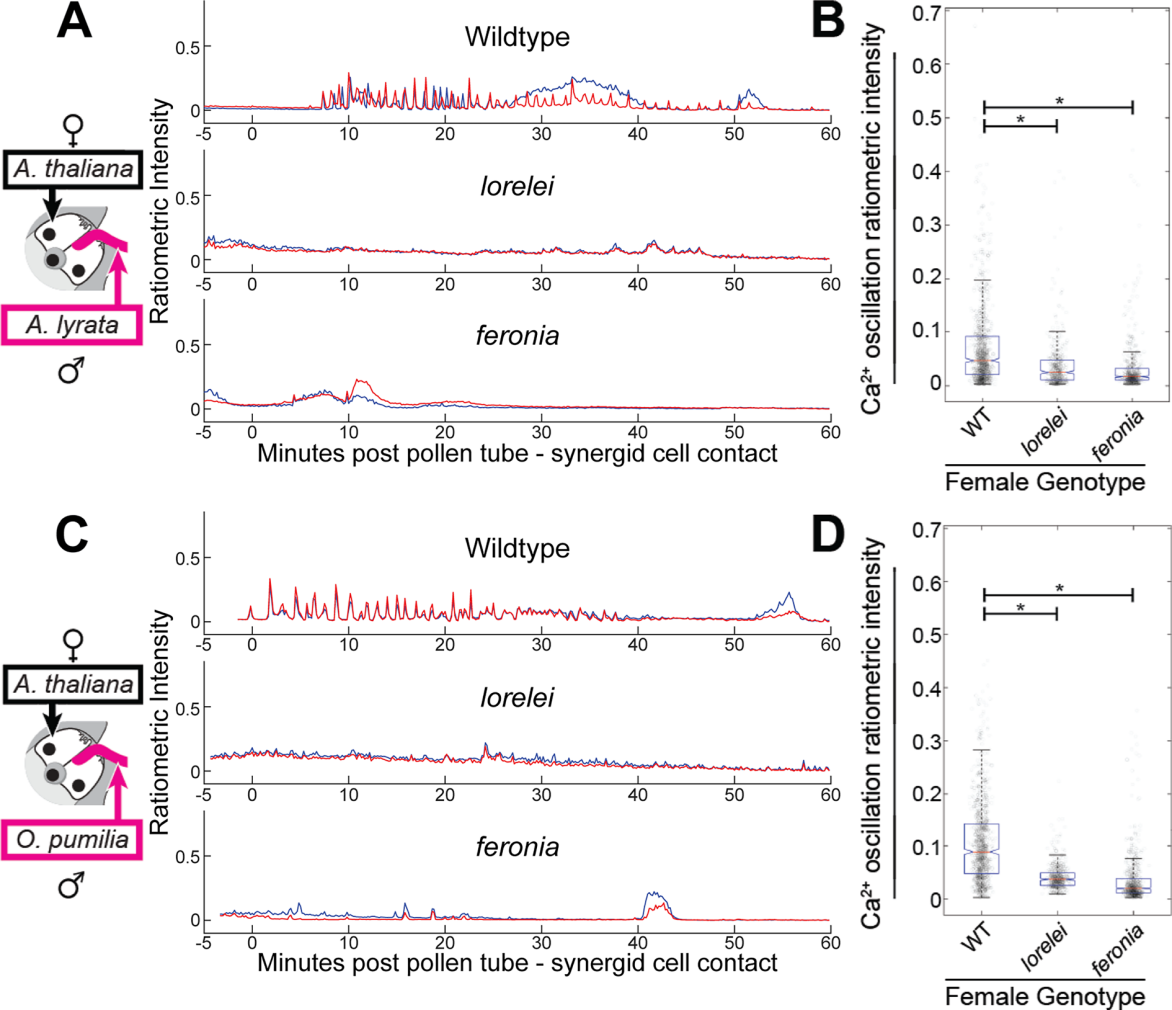
Interspecific pollen tubes elicit FER/LRE-dependent calcium ion oscillations in wild-type synergid cells. **A** Example traces from crosses onto wild-type, *lre*, and *fer* mutant females using wild-type *Arabidopsis lyrata* pollen. time point 0 indicates the moment of pollen tube–synergid cell contact. **B** Plot of ratiometric intensity of individual calcium ion oscillations observed during crosses onto female genotypes (wild type, *n* = 10 traces, *n* = 947 peaks; *lre* *n* = 10 traces, *n* = 398 peaks; *fer,* *n* = 10 traces, *n* = 480 peaks). Statistical significance (*) calculated using analysis of variance (ANOVA, α = 0.05). **C** Example traces from crosses onto wild-type, *lre*, and *fer* mutant females using wild-type *Olimarabidopsis pumila* pollen. Time point 0 indicates the moment of pollen tube–synergid cell contact. **D** Plot of calcium peaks (as in B) observed during crosses onto female genotypes (wild type, *n* = 10 traces, *n* = 912 peaks; *lre,* *n* = 10 traces, *n* = 461 peaks; *fer,* *n* = 10 traces, *n* = 589 peaks). Boxplots defined as in Fig. 2G. Statistical significance calculated using analysis of variance (ANOVA, α = 0.05)

During crosses using either *Arabidopsis lyrata* (*n* = 10) or *Olimarabidopsis pumila* (*n* = 10) males and wild-type *Arabidopsis thaliana* females, it was observed that interspecific pollen tubes were able to induce calcium ion oscillations in synergid cells, but pollen tube burst was not observed in any interspecific cross, indicating that induction of calcium ion oscillations alone is not sufficient to induce pollen tube lysis. (Fig. 3A, C, Supplementary Figs. 2A, 3A, Supplementary Videos 4, 7). These results suggest that *A. lyrata* and *O. pumila* pollen tubes are able to successfully signal their arrival to female cells of a different species, but that successful sperm release requires congruity in a distinct molecular recognition event that follows initiation of calcium oscillations.

To investigate whether the calcium ion oscillations observed during interspecific crosses are FER/LRE signaling complex-dependent, *A. lyrata* or *O. pumila* pollen was crossed onto *A. thaliana lre* or *fer* females. If the calcium ion oscillations observed during interspecific pollinations are dependent on FER/LRE, as they are during same-species pollination, then an attenuation of calcium ion responses during interspecific crosses onto either *fer *or *lre *females would be expected ((Ngo et al. 2014), Fig. 2F, G). Indeed, an attenuation of calcium ion oscillations in *lre* and *fer* mutant females receiving interspecific pollen tubes was observed (Fig. 3A–D, Supplementary Fig. 2B, C, Supplementary Fig. 3B, C, Supplementary Videos 5, 6, 8, 9), similar to results previously reported for *lre *and *fer* mutants receiving wild-type *A. thaliana* pollen tubes ((Ngo et al. 2014), Fig. 2, Supplementary Fig. 1, Supplementary Videos 2, 3). These results suggested that FER and LRE are required for the interspecific pollen tube-induced calcium ion oscillations, and a failure to present congruous identification signals to the receptive synergid cell occurs downstream of FER/LRE activation, or through an independent parallel mechanism.

### myb97, 101, 120 pollen tube mutants elicit FER/LRE-dependent calcium oscillations

Next, the possibility that the pollen tube is the source of signaling molecules underlying recognition by the synergid cell was explored. For this purpose, the *myb97, 101, 120* transcription factor triple mutant that severely downregulates pollen tube signaling components crucial for pollen tube–synergid cell signaling was used as a pollen donor for same-species crosses. *myb97, 101, 120* triple mutants lack crucial signaling components including Rapid Alkalinization Factor (RALF) 6, 7, 16, 36, and 37, which have been shown to bind FER, HERK, and ANJ, and are required for successful pollen tube burst (Zhong et al. 2022). Given the similarity in pollen tube overgrowth phenotypes observed during interspecific pollinations and *myb97, 101, 120* mutant pollinations (Leydon et al. 2014), it was hypothesized that the MYB-dependent gene regulatory network, including the five above-mentioned RALFs, could constitute some portion of the genetic identity that must be properly perceived during pollen tube–synergid signaling.

**Fig. 4:**
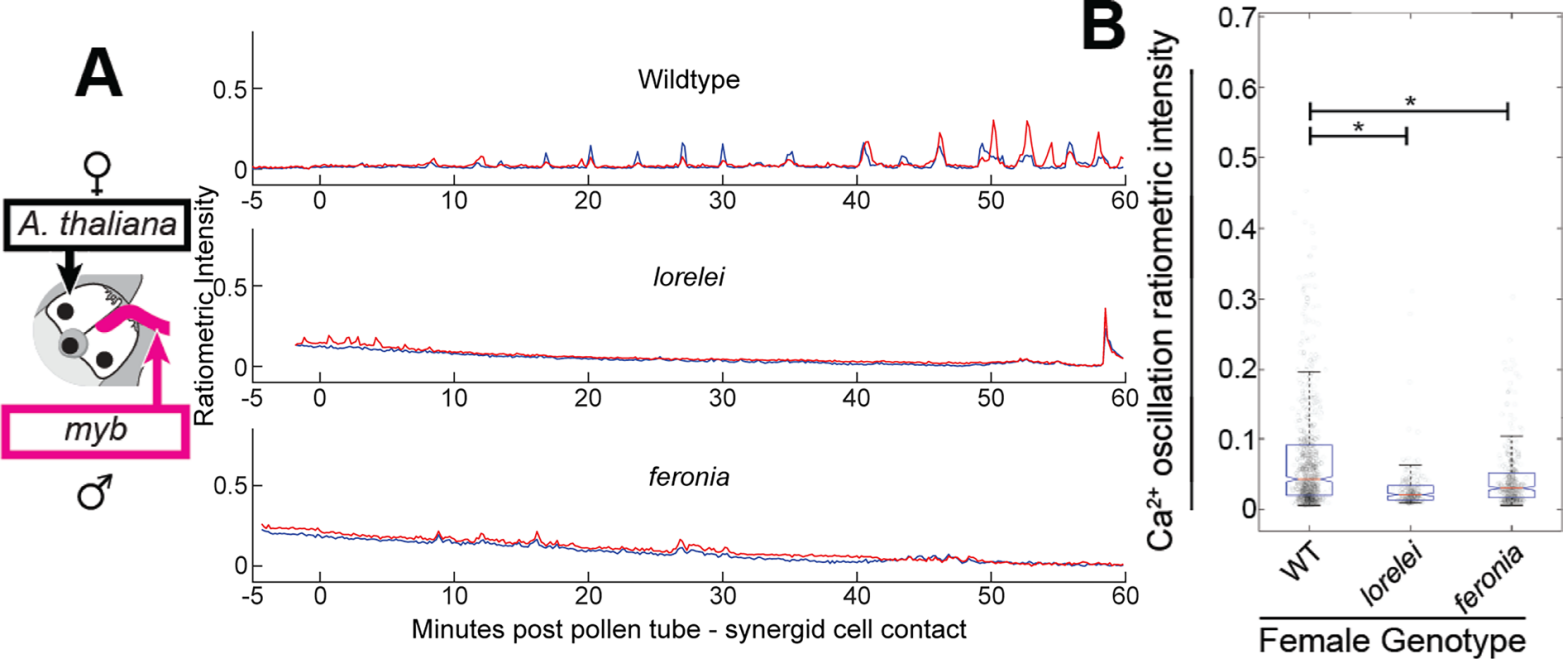
*myb 97, 101, 120* pollen tubes elicit FER/LRE-dependent calcium ion oscillations in wild-type synergid cells. **A** Example traces from crosses onto wild-type, *lre*, and *fer* mutant females using *myb97, 101, 120* triple-mutant *Arabidopsis thaliana* pollen. time point 0 indicates the moment of pollen tube–synergid cell contact. **B** Plot of ratiometric intensity of individual calcium ion oscillations observed during crosses onto female genotypes (wild type, *n* = 10 traces, *n* = 689 peaks; *lre,* *n* = 10 traces, *n* = 222 peaks; *fer,* *n* = 10 traces, *n* = 343 peaks). Boxplots defined as in Fig. 2G. Statistical significance calculated using analysis of variance (ANOVA, α = 0.05)

When *myb97, 101, 120* pollen was crossed to wild-type *A. thaliana* pistils, pollen tubes elicited calcium ion oscillations within synergid cells (Fig. 4A, Supplementary Fig. 3A, Supplementary Video 10) but still failed to burst, suggesting that MYB factors may play multiple roles during pollen tube reception via interactions with, or in parallel to, the roles of FERONIA/LORELEI. To assess whether the calcium ion oscillations induced by the *myb* triple-mutant required FER and LRE, same-species crosses between *myb* triple mutant pollen and *lre* and *fer *mutant females were performed. It was observed that, as in crosses with wild-type same-species pollen tubes, and interspecific pollen tubes, calcium ion oscillations are severely attenuated in *lre* and *fer *mutant females interacting with *myb97, 101, 120* mutant pollen tubes (Fig. 4A, B, Supplementary Fig. 4B, C, Supplementary Videos 11, 12). In the majority of crosses (8/10 in *lre* x *myb* and 6/10 in *fer* x *myb*), calcium transients were entirely ablated with the remaining fraction displaying an occasional calcium ion transient (Supplementary Fig. 4B, C). In such cases, calcium ion transients persisted briefly and were, on average, less prominent than in crosses onto wild-type females (Fig. 4B). These results suggested that the *myb97, 101, 120* triple mutant pollen tubes are able to successfully trigger LRE/FER-dependent synergid cell calcium ion oscillations despite severe downregulation of MYB-dependent RALFs (FER ligands, (Zhong et al. 2022)).

### Calcium ion oscillation frequency and intensity are not the sole determining factors for successful pollen tube–synergid rupture

**Fig. 5:**
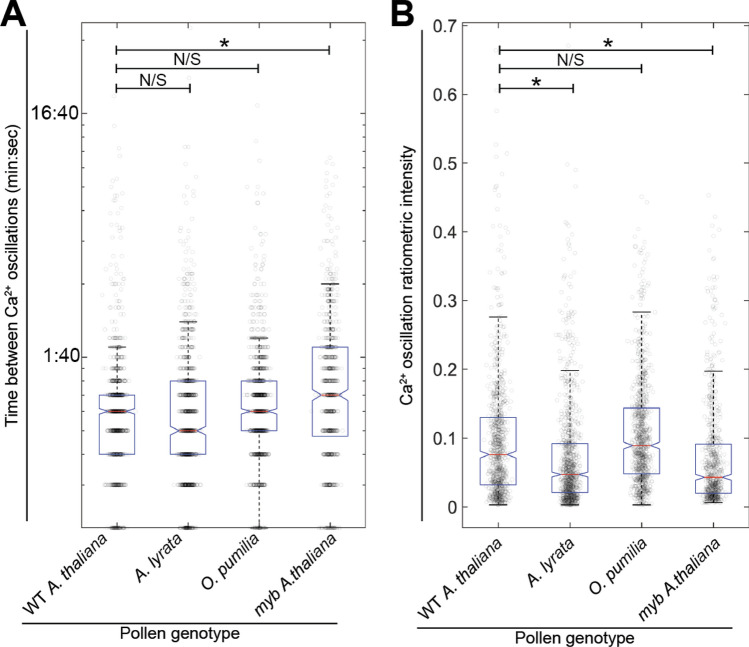
Calcium ion oscillation frequency and intensity vary in synergid cells interacting with *myb* mutant pollen tubes and in synergid cells interacting with interspecific pollen tubes. **A** Analysis of calcium ion oscillation frequency. Time between subsequent calcium ion transients was calculated and plotted on y-axis (circles). Y-axis displayed using log scale, as the majority of transients occur within three minutes of one another. **B** Analysis of ratiometric calcium ion oscillation amplitude. Amplitude of transients was determined by the ratiometric intensity of YFP/CFP fluorescence and plotted on y-axis (circles). Boxplots defined as in Fig. 2G. Statistical significance calculated using analysis of variance (ANOVA, α = 0.05)

Next, it was asked whether differences in the patterns of calcium ion oscillations recorded from wild-type *Arabidopsis thaliana* synergid cells responding to intraspecific, interspecific, or *myb* mutant pollen tubes could be discerned. Interactions between plants and fungal or bacterial symbionts initiate calcium ion oscillations in root hair cells, and it has been proposed that the frequency of the oscillations depends on the species identity of the symbiont (Charpentier et al. 2016; Wais et al. 2002; Kosuta et al. 2008). When the frequency of the calcium ion oscillations induced by pollen tubes in this dataset was examined, it was found that *Arabidopsis lyrata* and *Olimarabidopsis pumila* tubes induce oscillations with similar frequency to those induced by same-species pollen tubes (Fig. 5A). However, the calcium ion oscillations induced by *myb* mutant pollen tubes occur with a lower frequency than those induced by wild-type *Arabidopsis thaliana* pollen tubes (Fig. 5A, Supplementary Fig. 5). While no link between the frequency of the calcium ion oscillations and interspecific identity was observed in this system, it is interesting that the *myb* triple mutant induces calcium ion oscillations with lower frequency, suggesting that the expression level of signaling molecules may be tied to the frequency of calcium ion response.

Next, the amplitude of the calcium ion oscillation (Fig. 5B) and whether amplitude is tied to species identity was examined. It was found that on average the calcium ion oscillations induced by *Arabidopsis lyrata* and *myb* triple mutant pollen tubes were less intense than those induced by wild-type *Arabidopsis thaliana* pollen tubes. However, the calcium ion oscillations induced by *Olimarabidopsis pumila* pollen tubes were not significantly different from those induced by wild-type *A. thaliana.* Many roles of calcium ion oscillations have been proposed for different cell types, including the recognition of species identity. Here, it was found that the overall patterns of calcium ion oscillations varied between crossing schemes but, as with calcium ion frequency, the amplitude of the calcium ion oscillation does not seem to be directly tied to the success of pollen tube–synergid cell signaling.

## Discussion

In this study, calcium ion oscillations induced in the cytoplasm of synergid cells responding to the arrival of pollen tubes of either an exotic species (*A*. *lyrata* or *O. pumila*) or the same species (*A. thaliana* wild-type or *myb97, 101, 120* triple mutant) were recorded and examined. A simple naming scheme for each synergid (left or right) was used in this study, based on their relative position during observation. It was not possible to ascribe receptive v. non-receptive synergids (as in Ngo et al. 2014) in the experiments described in the present study because in many cases neither the pollen tube nor the synergid ruptured. Moreover, it was found that the pollen tube was often in contact with both synergid cells simultaneously, oscillations occurred in both synergid cells, and the large increase in calcium ion intensity associated with pollen tube burst occurred, at least occasionally, in the non-receptive synergid cell (as in Fig. 1). Importantly, it was found that in all cases the initiation of calcium ion oscillations was dependent on the FER/LRE signaling complex. The amplitude of calcium ion oscillations between crossing schemes was variable, but does not appear to be explicitly linked to pollen tube–synergid cell signaling success or pollen tube burst and release of sperm cells. During crosses with *myb* mutant pollen (which fail to release sperm), calcium ion oscillations occurred with a lower frequency than during wild-type crosses, but interspecific pollen tubes (which also fail at high rates to release sperm) had no significant effect on calcium ion oscillation frequency, suggesting that the frequency of calcium ion oscillations is not indicative of a successful interaction in this system. It was previously shown using pollinated pistil imaging that interspecific pollen tubes and *myb* triple-mutant pollen tubes successfully burst and fertilize ovules at low rates (Leydon et al. 2014). However, in the live imaging experiments used in the present study, *myb* triple-mutant or interspecific pollen tube burst was not observed. This is likely due to suboptimal pollen tube growth and/or signaling conditions imparted by live excision and single-cell confocal microscopy, which exacerbate incongruous mutant or interspecific pollen-pistil interactions. Future experimental systems (e.g., Desnoyer and Grossniklaus 2023) optimizing cell–cell interactions and facilitating acquisition of greater numbers of replicates will be important to further dissect the differences between signaling events that precede successful or unsuccessful interactions using interspecific or mutant pollen donors. The pollen tube MYB transcription factors regulate the expression of FER ligands (Leydon et al. 2017; Zhong et al. 2022) and the *myb* triple-mutant observations from this study suggest the degree to which pollen tube signaling peptides are expressed may affect the rate of synergid cell calcium ion oscillations. These data agree with observations that showed pollen tube-potentiated activation of synergid-expressed NTA must overcome Calmodulin-mediated NTA repression to proceed through phases of pollen tube reception via NTA’s function as a calcium channel (Gao et al. 2022).

Previous reports (Escobar-Restrepo et al. 2007) suggested that FER may be involved in a species-specific process that scrutinizes the pollen tube’s genetic identity. The data presented here suggest that pollen tube–synergid cell signaling defects during interspecific crosses, and during crosses using *myb97, 101, 120* mutant pollen, are not due to a failure to activate the FER/LRE signaling complex. Rather, interspecific and *myb* mutant pollen are defective in signaling processes that occur downstream of, or in parallel to FER/LRE-dependent calcium ion oscillations. FER mediates multiple signaling processes at the signaling interface that all contribute to cellular communication between the pollen tube and synergid cell. In addition to the initiation of calcium ion oscillations, FER is required for the establishment of ROS accumulation that is crucial for pollen tube rupture (Duan et al. 2014) and it has been observed that mutations in *herk* and *anj* phenocopy the pollen tube overgrowth observed in *fer* mutants, but do not affect the FER-dependent accumulation of ROS at the pollen tube–synergid cell interface (Galindo-Trigo et al. 2020). Previously, it was also shown that the relocalization of NTA relies on FER/LRE signaling (Kessler et al. 2010). Recent studies have found that a NTA transgene constitutively localized to the filiform apparatus reduces pollen tube coiling defects during interspecific crosses to a greater degree than wild-type NTA, but only when FER is also present at the interface (Ju et al. 2021). It was proposed that FER/LRE may both direct NTA localization and also provide a priming signal to the arriving pollen tube that is crucial during interspecific crosses for the subsequent function of NTA as a calcium ion channel (Gao et al. 2022). In agreement with this proposition, the data from the present study suggest the possibility that a lack of adequate pollen tube potentiation subsequent to FER/LRE activation could preclude successful burst.

Four members of the CrRLK1L family have overlapping functions that control pollen tube integrity (BUPS1, BUPS2, ANX1, ANX2); loss of function of these genes results in premature pollen tube rupture (Boisson-Dernier et al. 2009; Ge et al. 2017; Mecchia et al. 2017). Recently, it was shown that reduction of BUPS1 function caused pollen tubes to burst as they exited the confined intercellular space of the style and entered the more open transmitting tissue (Zhou et al. 2021). This observation led to the finding that BUPS1 is required to activate pollen tube tip-localized ROP GTPase activity in response to changes in pollen tube mechanical pressure (Zhou et al. 2021). These findings suggest that BUPS1 (and other CrRLK1L family members) could act as mechanosensors, with activities that are potentiated by cell-type specific RALF ligands (Zhou et al. 2021). Taken with the finding that FER is required for mechanically induced calcium oscillations in cells of Arabidopsis roots (Shih et al. 2014), this suggests the possibility that mechanical deformation commonly observed as pollen tubes and synergid cells interact (e.g., Supplementary Videos 1–12) could be involved in contributing to FER/LRE/NTA activation, and/or potentiation of pollen tube bursting.

Using the calcium ion oscillation signature as a readout for points of failure during pollen tube–synergid cell signaling has clarified the role of this process as a prezygotic barrier to interspecific hybridization. Sperm release fails in crosses between closely related species and it was observed here that pollen tube-induced, FER/LRE-dependent calcium ion oscillations occur in interspecific crosses but are insufficient to induce sperm release. Future work aimed at visualizing NTA movement dynamics, calcium ion oscillations, and ROS homeostasis simultaneously during interspecific pollen tube–synergid cell signaling will further clarify the molecular signals required for interspecific congruence during downstream phases of pollen tube reception.

## Materials and methods

### Transgenic lines

Wild-type and *lre* mutant background pMYB98:YC3.60 (Nagai et al. 2004) lines were supplied by Dr. Ravishankar Palanivelu’s Laboratory at the University of Arizona. pMYB98:YC3.60 was introgressed into the *fer-4* mutant background which were gifted by Dr. Sharon Kessler’s Laboratory at Purdue University. The *Arabidopsis thaliana* pLAT52:dsRed line was gifted by Dr. Gregory Copenhaver’s Laboratory at The University of North Carolina. *Olimarabidopsis pumila* was transformed with pLAT52:dsRed using Agrobacterium-mediated transformation by Dr. Alexander Leydon (Current address: Department of Biology; University of Washington, Seattle). The *myb97, 101, 120* was generated by Dr. Alexander Leydon. All genotyping primer sequences used for this study are available upon request.

### Arabidopsis growth, pistil dissection, and time lapse microscopy

Arabidopsis plants were grown under 16-h-light, 8-h-dark until mature. Pistils were emasculated 24 h before dissections. Pistils were pollinated and left for 45 min before dissection. 200 µl of Arabidopsis pollen growth media (5 mM KCl, 0.01% H3BO3, 5 mM CaCl2, 1 mM MgSO4, 10% Sucrose, 1.5% NuSeive GTG Agarose, pH 7.6) was allowed to cool as a pad in a MatTek 35-mm glass-bottom dish with No. 1.5 coverglass (part no. P35G-1.5–20-C, https://www.mattek.com/store/p35g-1-5-20-c-case/). After media had cooled, pistils were dissected such that the valves were removed, and the two opposing edges of the replum were left attached to the underside of the style. The transmitting tract was split from the bottom up, and the two rows of ovules were carefully pulled away from one another and laid out in opposing directions onto media. The author’s found that leaving the transmitting tract/replum attached to the underside of the style (in an arrangement referred to as Further-*In-Vivo* (FIV), described above, Supplementary Video 13) led to higher numbers of successful vs. unsuccessful interactions observed during imaging (data not shown). “FIV” preps were left in a humidity chamber at 22 °C for 5 h. Dishes were imaged on a Zeiss LSM Laser Scanning Confocal Microscope at 20 × optical zoom with 3.5 × digital zoom. Images were taken with a 10 s interval starting before pollen tube contact with synergid cells and continuing for 1 h after contact. For *Arabidopsis lyrata*, because pollen-expressed fluorescent reporters in this species were not available, it was not possible to track the growth of the pollen tube tip after it had grown through the micropyle. To address this, during wild-type *A. thaliana* pMYB98:YC3.60 × *A. lyrata* crosses, the initial calcium ion transient observed in synergid cells was used as the marker for contact between the pollen tube tip and synergid cell. During *lre* pMYB98:YC3.60 × *A. lyrata* crosses and *fer* pMYB98:YC3.60 × *A. lyrata* crosses, the deformation of the synergid cell was used as the marker for contact between the pollen tube tip and synergid cell, as in these mutant backgrounds calcium ion oscillations are largely abolished^4^. For crosses using *A. thaliana* or *O. pumila* pollen donors, pollen-expressed red fluorescence allowed us to track the pollen tube as it grew into the embryo sac and first contact was defined visually.

### Time lapse data set analysis

To analyze time lapse datasets, a custom ImageJ (https://imagej.nih.gov/ij/) macro (supplied in Supplementary File 4 as a commented, editable.ijm file) was used. Any time lapse movies suffering from drift were corrected using the MultiStackReg plugin (http://bradbusse.net/sciencedownloads.html). Supplementary File 4 splits multi-channel time lapse datasets into constituent channels, asks the user to draw regions of interest around the left and right synergid cells, subtracts any pollen-expressed fluorescence from synergid cell channels, and uses the RatioPlus plugin (https://imagej.nih.gov/ij/plugins/ratio-plus.html) to calculate relative FRET efficiency within the regions of interest. Following this, bright outliers were removed, and the images were despeckled to remove noise resulting from our data collection process. The ratiometric results are saved automatically as.csv files, along with the summed projection over which the user draws the regions of interest around the synergid cells, the coordinates of regions of interest themselves, and a flattened composite time lapse to the directory from which the time lapse dataset is opened.

### FRET data analysis

Data were analyzed using MATLAB (available at https://www.mathworks.com/products/matlab.html). The MATLAB script used to concatenate fluorescent data (Supplementary File 1) was written by NDP and is supplied as Supplementary File 5. The MATLAB script used to plot concatenated data as colored lines was written by NDP and is provided as Supplementary File 6. The MATLAB script used to plot concatenated data as boxplots was written by NDP and is supplied as Supplementary File 7. Supplementary File 7 relies on the ViolinPlot repository (available at https://github.com/bastibe/Violinplot-Matlab). For Figs. 1, 2 and 3, calcium ion peaks were analyzed if they were greater than 1.1 × the minimum prominence value registered for their respective trace. This cutoff value was chosen such that noise could be excluded from the analysis while still allowing traces with few/small prominences (e.g., those onto *lre* or *fer* females) could still be analyzed. For Fig. 4, calcium ion peaks with prominences greater than 5 × the minimum prominence value registered for their respective traces were analyzed.

### Data plotting and figure construction

For each cross scheme all ten replicates were sorted according to average calcium peak prominence. Prominence was defined as the height of the calcium ion transient with respect to the nearest trough separating the transient from a transient with equal or greater height. This method of sorting the traces was chosen because, while the varying length of oscillations between traces affected the number of transients that could be used to calculate the average prominence, it was not expected to have an effect on the averaging beyond this. Traces shown in Figs. 1, 2 and 3 were chosen as they are the trace with median peak prominence. For each statistical plot in Figs. 1, 2, 3 and 4, the median value was denoted by notch. 95% confidence interval about the median is denoted by notches. Box defines interquartile range. Length of whiskers is calculated as 1.5*IQR. ANOVA and post-hoc multiple comparison of means supplied as Supplementary Files 2 and 3.

#### Author contribution statement

NDP and MAJ designed and planned the experiments. NDP performed the experiments and collected the data. NDP analyzed the data. NDP and MAJ wrote the manuscript.

### Supplementary Information

Below is the link to the electronic supplementary material.

Supplementary Figure 1. A. 10 replicate timelapse calcium ion traces of pMYB98:YC3.60 in wild type Arabidopsis thaliana crossed as female with wild type Arabidopsis thaliana pollen. B. 10 replicate timelapse calcium ion traces of pMYB98:YC3.60 in lre mutant background Arabidopsis thaliana crossed as female with Wildtype Arabidopsis thaliana pollen. C. 10 replicate timelapse calcium ion traces of pMYB98:YC3.60 in fer mutant background Arabidopsis thaliana crossed as female with Wildtype Arabidopsis thaliana pollen. For each trace, the left synergid region of interest is plotted with a blue line and the right synergid region of interest is plotted with a red line. Red dot: traces shown in Figure 2. For each trace, time point 0 indicates the moment of pollen tube - synergid cell contact. For (A), pollen tube - synergid cell burst is indicated with black asterisk (TIF 77178 kb)

Supplementary Figure 2. A. 10 replicate timelapse calcium ion traces of pMYB98:YC3.60 in Wildtype Arabidopsis thaliana crossed as female with Wildtype Arabidopsis lyrata pollen. B. 10 replicate timelapse calcium ion traces of pMYB98:YC3.60 in lre mutant background Arabidopsis thaliana crossed as female with Wildtype Arabidopsis lyrata pollen. C. 10 replicate timelapse calcium ion traces of pMYB98:YC3.60 in lre mutant background Arabidopsis thaliana crossed as female with Wildtype Arabidopsis lyrata pollen. For each trace, the left synergid region of interest is plotted with a blue line and the right synergid region of interest is plotted with a red line. For each trace, time point 0 indicates the moment of pollen tube - synergid cell contact. Red dot: traces shown in Figure 3 (TIF 77148 kb)

Supplementary Figure 3. A. 10 replicate timelapse calcium ion traces of pMYB98:YC3.60 in Wildtype Arabidopsis thaliana crossed as female with Wildtype Olimarabidopsis pumila pollen. B. 10 replicate timelapse calcium ion traces of pMYB98:YC3.60 in lorelei-mutant background Arabidopsis thaliana crossed as female with Wildtype Olimarabidopsis pumila pollen. C. 10 replicate timelapse calcium ion traces of pMYB98:YC3.60 in fer mutant background Arabidopsis thaliana crossed as female with Wildtype Olimarabidopsis pumila pollen. For each trace, the left synergid region of interest is plotted with a blue line and the right synergid region of interest is plotted with a red line. For each trace, time point 0 indicates the moment of pollen tube - synergid cell contact. Red dot: traces shown in Figure 3 (TIF 77352 kb)

Supplementary Figure 4. A. 10 replicate timelapse calcium ion traces of pMYB98:YC3.60 in wild-type Arabidopsis thaliana crossed as female with myb97,101,120 triple-mutant Arabidopsis thaliana pollen. B. 10 replicate timelapse calcium ion traces of pMYB98:YC3.60 in lre mutant background Arabidopsis thaliana crossed as female with myb97,101,120 triple-mutant Arabidopsis thaliana pollen. C. 10 replicate timelapse calcium ion traces of pMYB98:YC3.60 in fer mutant background Arabidopsis thaliana crossed as female with myb97,101,120 triple-mutant Arabidopsis thaliana pollen. For each trace, the left synergid region of interest is plotted with a blue line and the right synergid region of interest is plotted with a red line. Red dot: traces shown in Figure 4 (TIF 77099 kb)

Supplementary Figure 5. A. 10 replicate timelapse calcium ion traces of pMYB98:YC3.60 in wild-type Arabidopsis thaliana crossed as female with wild-type Arabidopsis thaliana pollen. B. 10 replicate timelapse calcium ion traces of pMYB98:YC3.60 in wild type Arabidopsis thaliana crossed as female with myb97,101,120 triple-mutant Arabidopsis thaliana pollen. C. 10 replicate timelapse calcium ion traces of pMYB98:YC3.60 in wild-type Arabidopsis thaliana crossed as female with wild-type Arabidopsis lyrata pollen. For each trace, the left synergid region of interest is plotted with a blue line and the right synergid region of interest is plotted with a red line. D. 10 replicate timelapse calcium ion traces of pMYB98:YC3.60 in wild-type Arabidopsis thaliana crossed as female with wild-type Olimarabidopsis pumila pollen. For each trace calcium ion peaks that were used for frequency analysis (with a prominence greater than 5x minimum prominence observed) are marked with dots (TIF 104173 kb)

Supplementary Video 1: Wildtype *A. thaliana* pMYB98:YC3.60 x Wildtype *A. thaliana* time-lapse movie (AVI 4066 kb)

Supplementary Video 2: *Ire* mutant *A. thaliana* pMYB98:YC3.60 x Wildtype *A. thaliana* time-lapse movie (AVI 4057 kb)

Supplementary Video 3: *fer* mutant *A. thaliana* pMYB98:YC3.60 x Wildtype *A. thaliana* time-lapse movie (AVI 2128 kb)

Supplementary Video 4: Wildtype A. thaliana pMYB98:YC3.60 x Wildtype *A. lyrata* time-lapse movie (AVI 5901 kb)

Supplementary Video 5: *Ire* mutant *A. thaliana* pMYB98:YC3.60 x Wildtype *A. lyrata* time-lapse movie (AVI 6949 kb)

Supplementary Video 6: *fer* mutant *A. thaliana* pMYB98:YC3.60 x Wildtype *A. lyrata* time-lapse movie (AVI 5506 kb)

Supplementary Video 7: Wildtype *A. thaliana* pMYB98:YC3.60 x Wildtype *O. pumila* time-lapse movie (AVI 2897 kb)

Supplementary Video 8: *Ire* mutant *A. thaliana* pMYB98:YC3.60 x Wildtype *O. pumila* time-lapse movie (AVI 4137 kb)

Supplementary Video 9: *fer* mutant *A. thaliana* pMYB98:YC3.60 x Wildtype *O. pumila* time-lapse movie (AVI 3716 kb)

Supplementary Video 10: Wildtype *A. thaliana* pMYB98:YC3.60 x *myb97,101,120* *A. thaliana* time-lapse movie (AVI 5958 kb)

Supplementary Video 11: *Ire* mutant *A. thaliana* pMYB98:YC3.60 x *myb97,101,120* *A. thaliana* time-lapse movie (AVI 3933 kb)

Supplementary Video 12: *fer* mutant *A. thaliana* pMYB98:YC3.60 x *myb97,101,120* *A. thaliana* time-lapse movie (AVI 4048 kb)

Supplementary Video 13: Dissection approach for “Further-*In-Vivo*” preparation (AVI 175726 kb)

## Data Availability

Raw data for the following figures are provided as listed. All original confocal time lapse datasets and region of interest coordinates are available upon request from https://doi.org/10.26300/b6za-nt53. Figure 1, 2 and 3—Raw trace data provided in Supplementary File 1. Figure 1, 2, 3 – Raw statistical data provided in Supplementary File 2.

## Code availability statement

All code used in this manuscript is supplied as supplementary data. Data for this study was collected using Zen Black (available through Zeiss), and the open-source ImageJ platform (available at https://imagej.nih.gov/ij). Ratiometric calculations were performed using a custom ImageJ.IJM file (Supplementary File 4) written by NDP that depends on the RatioPlus plugin (available at https://imagej.nih.gov/ij/plugins/ratio-plus.html). Data were analyzed using MATLAB (available at https://www.mathworks.com/products/matlab.html). The MATLAB script used to organize fluorescent data for plotting was written by NDP and is supplied as Supplementary File 5. The MATLAB script used to plot concatenated data as colored lines was written by NDP and is provided as Supplementary File 6. The MATLAB script used to plot concatenated data as boxplots was written by NDP and is supplied as Supplementary File 7. Supplementary File 7 relies on the ViolinPlot repository (available at https://github.com/bastibe/Violinplot-Matlab).
